# What is the recommended procedure for recurrent rectal prolapse? A retrospective cohort study in a single Japanese institution

**DOI:** 10.1007/s00595-020-02190-5

**Published:** 2021-01-09

**Authors:** Kimihiko Funahashi, Akiharu Kurihara, Yasuyuki Miura, Mitsunori Ushigome, Tomoaki Kaneko, Satoru Kagami, Yu Yoshino, Takamaru Koda, Yasuo Nagashima, Kimihiko Yoshida, Yu Sakai

**Affiliations:** Department of Gastroenterological Surgery, Toho University Omori Medical Center, 6-11-1 Omorinishi, Otaku, Tokyo, 143-8541 Japan

**Keywords:** Recurrent rectal prolapse, Laparoscopic suture rectopexy, Perineal procedure, Choice of surgical treatment

## Abstract

**Purpose:**

The choice of surgical procedure for rectal prolapse (RP) is challenging because of the high recurrence and morbidity rates. We aimed to clarify whether laparoscopic suture rectopexy (lap-rectopexy) is suitable for Japanese patients with recurrent RP.

**Methods:**

We retrospectively evaluated 77 recurrent RP patients who had been treated on average 1.5 times between June 2008 and April 2016. Forty-one patients underwent lap-rectopexy and 36 underwent perineal procedures. We compared surgical outcomes and recurrence rate following surgery between the two groups. The multivariable logistic regression analysis was performed to determine risk factors of recurrent RP.

**Results:**

In patients’ characteristics, significant differences were observed in the type of anesthesia (*p *< 0.01) and length of recurrent RP (*p *= 0.030). The mean operative time was significantly longer in the lap-rectopexy group (*p *< 0.001). Blood loss, length of hospitalization, and postoperative complications were similar. The recurrence rate was significantly lower in the lap-rectopexy group (17.1% vs. 38.9%, *p *= 0.032). Multivariate analysis showed that only the laparoscopic approach was significantly associated with a low recurrence following surgery (odds ratio 0.273, 95% CI − 2.568 to − 0.032).

**Conclusion:**

Lap-rectopexy is recommended for recurrent RP because its low recurrence rate and safety profile are similar to those of perineal procedures.

## Introduction

Rectal prolapse (RP) is a well-known, troublesome disease that develops mainly in older women. It is associated with significant morbidity, which may include rectal bleeding, obstructive defecation, and pain, resulting in reduced quality of life (QOL) of elderly patients with RP. Recently, RP has gained attention in Japan where the population is aging rapidly. Surgical interventions, including abdominal and perineal procedures, are usually required for RP. It is generally accepted that an abdominal procedure for RP has a lower recurrence rate and improved functional outcomes [1]. In contrast, perineal procedures—as typified by the Altemeier and Delorme procedures—have been performed widely because of its lower operative morbidity rate and good recovery after surgery for frail elderly patients with comorbidities [2]. However, multiple recurrences following surgical repair for RP is a critical problem in RP surgery. We often encounter patients with complaints of frequently recurring RP following surgery for RP. It can be challenging to decide the subsequent repair treatment that would be appropriate for patients with multiple recurrent RP following surgery. Recently, laparoscopic techniques have been added as treatment options for RP because of their advantages of early recovery, less pain, and the possibility of lower morbidity. Some researchers have reported that laparoscopic procedures represent a new surgical approach as an alternative to conventional abdominal procedures [3–8].

This retrospective study aimed to evaluate whether laparoscopic rectopexy (lap-rectopexy) is an appropriate treatment regarding surgical outcome and recurrence rate for patients experiencing recurrence following the surgical repair for RP.

## Methods

**Fig. 1:**
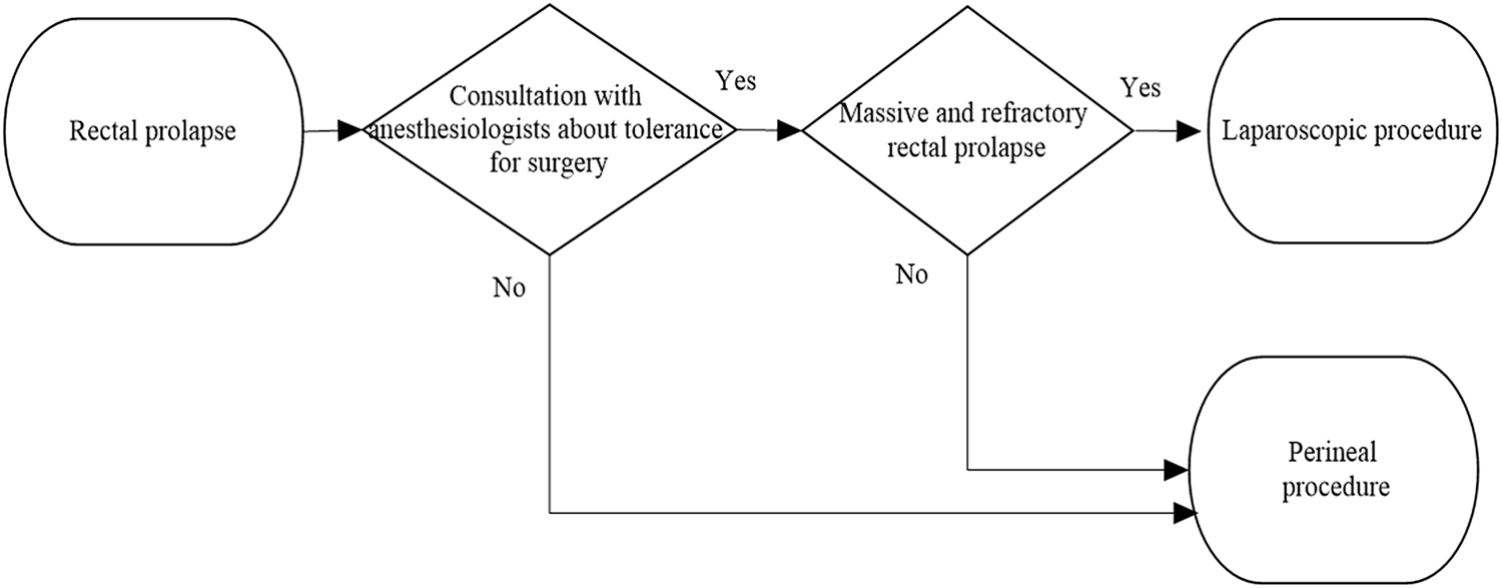
Algorithm in choosing surgical procedure for rectal prolapse

**Fig. 2:**
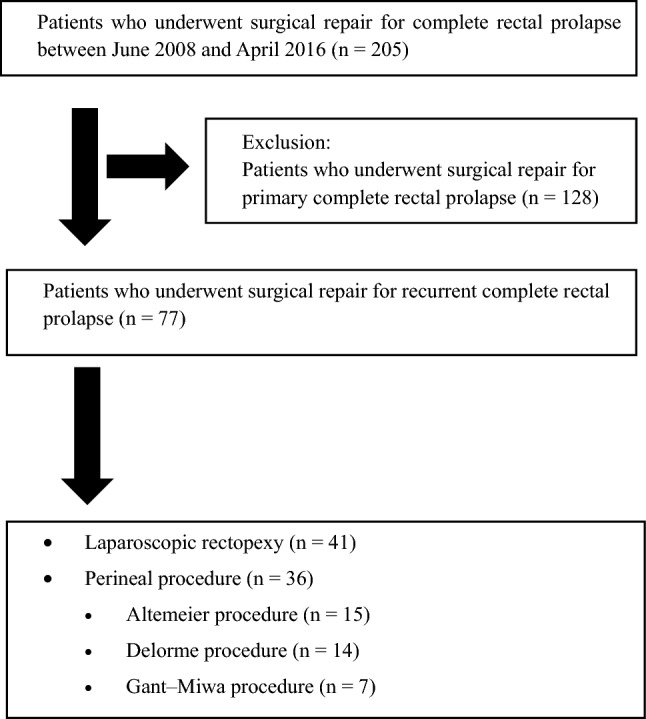
Patient selection

In total, 205 patients who underwent surgery for complete RP between June 2008 and April 2016 at Toho University Omori Medical Center were identified. For all patients with RP, tolerance for surgery, including frailty and comorbidities, was first evaluated before surgery by anesthesiologists. While referring to the anesthesiologists’ opinions, the procedure was selected based on the surgeon’s experience and/or preference (Fig. 1). RP patients with poor tolerance for surgery have been followed up with conservative management of bowel movement using laxatives. Of the 205 patients, 128 who underwent surgery for primary complete RP were excluded. Finally, we evaluated 77 patients who required surgical intervention for a complete recurrent RP; of these, 41 (53.2%) underwent lap-rectopexy (the lap-rectopexy group), and the remaining 36 underwent perineal procedures (the perineal procedure group), including the Altemeier procedure in 15 patients (19.5%), the Delorme procedure in 14 (18.2%), and other procedures in seven (9.1%) (Fig. 2). We compared the surgical outcomes, including operative time, blood loss, postoperative complications, and period of hospitalization, and recurrence following surgical repair for recurrent RP between the lap-rectopexy and perineal groups. Finally, multivariate analysis for risk factors of recurrence following surgery for recurrent RP was conducted between the lap-rectopexy and perineal procedure groups. The presence or absence of new recurrence was the dependent variable. In contrast, seven clinical variables, including sex, age [9], presence or absence of pelvic organ prolapse including uterine, vaginal, and bladder prolapse [10], number of surgical repairs previously underwent for RP [7], type of surgical repair previously underwent for RP (transabdominal vs. perineal approach), length of recurrent RP, and type of surgical approach for recurrent RP (lap-rectopexy vs. perineal approach) [1] were used as independent variables.

This retrospective cohort study was approved by the Toho University Omori Medical Center Ethics Committee (No. M19183). Informed consent was obtained from all individual participants included in this study.

### Statistical analysis

Comparison between the groups was performed using a *χ*^2^ test or Fisher’s exact test for categorical variables, and a Mann–Whitney *U* test for continuous variables. Tests of significance were two sided, and the values of *p *< 0.05 were considered statistically significant. The multivariable logistic regression analysis was performed to determine the risk factors of recurrent RP. All data were entered into a computer database and analyzed using the Statistical Package for the Social Sciences (SPSS) for Windows software program, version 9.0.2 (SAS Institute Inc., Cary, NC, USA).

## Results

### Patient characteristics

The patients’ characteristics are shown in Table 1. Female patients comprised 88.3% (*n* = 68) of the total population, and the patients’ median age was 80 years (range 17– 100 years). Eight patients (10.4%) had an ASA grade of 1, 57 (74.0%) had an ASA grade of 2, and 12 (15.6%) had an ASA grade of 3. Moreover, 31 patients (40.2%) had at least a comorbidity, including myocardial infarction, congestive heart disease, peripheral vascular disease, cerebrovascular accident, dementia, chronic obstructive pulmonary disease, chronic hepatitis, diabetes mellitus, chronic kidney disease, and solid tumor. Regarding previously underwent surgical repair for RP, 77.9% (*n* = 60) of 77 patients had previously undergone perineal procedures; of these, 16 patients (20.8%) previously underwent surgical repair for RP two or more times, and three underwent surgery five times or more. The median length of recurrent RP in the lap-rectopexy group was 50 mm (range 17–100 mm), which was significantly shorter than 60 mm (17–115 mm) in the perineal group (*p* = 0.030). Twenty-five patients (69.4%) in the perineal group underwent surgery under general anesthesia in this study *(p *< 0.01), and the median observation period was 972.5 days (range 8–3616 days).

**Table 1:** Patient characteristics

	Total (*n* = 77)	Laparoscopic rectopexy (*n* = 41)	Perineal procedure (*n* = 36)	*p* value
Sex, *n* (%)				0.835
Male	9 (11.7)	5 (12.2)	4 (11.1)	
Female	68 (88.3)	36 (87.8)	32 (88.9)	
Age, median (range)	80 (17–100)	80 (17–100)	80 (45–91)	0.094
ASA, *n* (%)				0.944
1	8 (10.4)	4 (9.8)	4 (11.1)	
2	57 (74.0)	31 (75.6)	26 (72.2)	
3	12 (15.6)	6 (14.6)	6 (16.7)	
Comorbidity, *n* (%)				0.818
Positive	31 (40.2)	17 (41.5)	14 (38.9)	
Negative	46 (59.8)	24 (58.5)	22 (61.1)	
Surgical repair previously underwent for RP, *n* (%)				0.351
Abdominal procedure	14 (18.2)	6 (14.6)	8 (22.2)	
Perineal procedure	60 (77.9)	34 (82.9)	26 (72.2)	
Unknown	3 (3.9)	1 (2.5)	2 (5.6)	
No. of surgical repairs previously underwent for RP, *n* (%)				0.294
Once	58 (75.3)	29 (70.7)	29 (80.6)	
Twice	9 (11.7)	7 (17.1)	2 (5.6)	
Three times	4 (5.2)	2 (4.9)	2 (5.6)	
≥ Four times	3 (3.9)	2 (4.9)	1 (2.6)	
Unknown	3 (3.9)	1 (2.4)	2 (5.6)	
Length of recurrent RP, mm (range)^a^	50 (17–115)	50 (17–100)	60 (17–115)	0.030
Anesthesia, *n* (%)				< 0.01
General	66 (85.7)	41 (100.0)	25 (69.4)	
Lumbar	11 (14.3)	0	11 (30.6)	
Observation period, day (range)^a^	972.5 (8–3616)	926.1 (10–3507)	972.5 (8–3616)	0.143

^a^Median, *ASA* American Society of Anesthesiologist, *RP* rectal prolapse

**Fig. 3:**
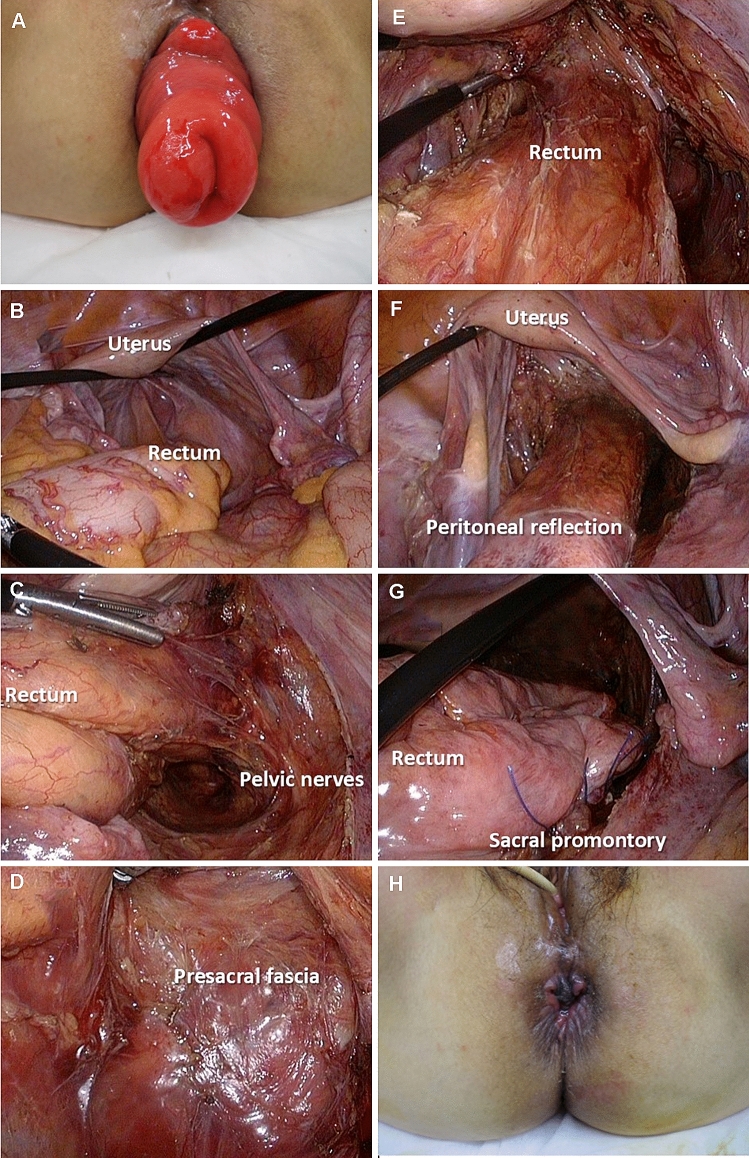
Technique of laparoscopic suture rectopexy. A patient with recurrent rectal prolapse following trans-perineal repair for rectal prolapse (**a**). A deep Douglas’ pouch is observed (**b**). We laparoscopically mobilized the rectum, preserving the pelvic nerves (**c**–**f**), and subsequently anchored the rectum at the sacral promontory using a few non-absorbable sutures (**g**). Final aspect (**h**)

Lap-rectopexy was performed without mesh for all patients. The rectum was dissected to the pelvic floor laparoscopically and subsequently directly anchored to the sacral promontory using a few non-absorbable sutures (Fig. 3). When we first started lap-rectopexy around 2012, mobilization of the rectum was performed laparoscopically, and the rectum was anchored using a few non-absorbable sutures while looking directly through a 4-cm small incision. This study included 19 patients who had undergone this procedure.

### Surgical outcomes

The surgical outcomes are shown in Table 2. The mean operative time was significantly longer in the lap-rectopexy group than in the perineal group [257.8 ± 110. 2 (mean ± standard deviation [SD]) min vs. 145.5 ± 51.6 min; *p *< 0.001]. The median blood loss was 20 mL (range 0–660 mL) in the lap-rectopexy group and 29 mL (range 0–798 mL) in the perineal group (*p* = 0.333). The median length of hospitalization was the same (15 days) in the two groups.

**Table 2:** Surgical outcomes

	Laparoscopic rectopexy (*n* = 41)	Perineal procedure (*n* = 36)	*p* value
Operative time, min (mean ± SD)	257.8 ± 110.2	145.5 ± 51.6	< 0.001
Blood loss, mL, median (range)	20 (0–660)	29 (0–798)	0.333
Postoperative complication, *n* (%)	7 (17.1)	4 (11.1)	0.675
I	1	1	
II	5	1	
III a	1	1	
III b	0	1	
IV	0	0	
Period of hospitalization, days, median	15	15	
Recurrence, *n* (%)	7 (17.1)	14 (38.9)	0.032
		Altemeier 4 (28.6)	
		Delorme 7 (50.0)	
		Gant-Miwa 3 (21.4)	
Period until recurrence following surgery, days, median (range)	120.0 (51–425)	84.5 (7–880)	0.332

*SD* standard deviation

No mortality was observed in this study, and postoperative complications were observed in 11 patients (14.3%). The postoperative complication rates were similar in both groups [seven patients (17.1%) in the lap-rectopexy group and four patients (11.1%) in the perineal group]. The postoperative complications of Grade 2 or more according to the Clavien–Dindo classification were more common in the lap-rectopexy group than in the perineal group (14.6% vs. 8.3%; *p* = 0.615). Two cardiac complications and one pulmonary complication were noted in the lap-rectopexy group.

### Recurrence

In this study, recurrence following surgical repair for recurrent RP was defined as the presence of complete RP on physical examination. Recurrence following surgical repair for recurrent RP occurred in 21 patients (27.3%): seven (17.1%) in the lap-rectopexy group and 14 (38.9%) in the perineal group. Of 14 patients who experienced recurrence in the perineal group, four cases (28.6%) occurred following the Altemeier procedure, seven (50.0%) following the Delorme procedure, and three (21.4%) following the Gant–Miwa procedure. Additionally, a significant difference was observed between the two groups (*p* = 0.032). The median period until recurrence was similar between the two groups (120 days in the lap-rectopexy group vs. 84.5 days in the perineal group, *p* = 0.332).

### Risk factors for recurrence

Seven clinical parameters were used as independent variables (Table 3). In the multivariable analysis, the recurrence rate following surgical repair for recurrent RP was significantly dependent on the particular approach used for the surgical repair of recurrent RP (odds ratio 0.273, 95% confidence interval − 2.568 to − 0.032, *p* = 0.046).

**Table 3:** Risk factors of recurrence

	*p*	95% confidence interval	Odds ratio
Sex	0.851	− 2.010 to 1.658	0.839
Age	0.985	0.038 to 0.039	1.000
Comorbidity of prolapse of other organs in the pelvis	0.909	−1.543 to 1.734	1.010
Type of previous surgical repair for RP (transabdominal/perineal)	0.514	− 1.932 to 0.967	0.617
No. of surgical repairs previously underwent for RP	0.694	− 1.162 to 1.746	1.339
Length of RP	0.143	− 0.007 to 0.050	1.021
Approach of surgical repair for recurrent RP (lap-rectopexy/perineal procedure)	0.046	− 2.568 to − 0.032	0.273

*RP* rectal prolapse

## Discussion

Healthy life expectancy has been emphasized since the World Health Organization advocated it in 2000. Healthy life expectancy in Japan has been the highest globally, while treatment for diseases affecting QOL of elderly patients is a serious social issue. RP, with morbidities such as rectal bleeding, obstructive defecation, and pain, often impair the activities of daily living in elderly patients. Recently, colorectal surgeons have been paying attention to RP, which develops mostly in elderly women, because the surgical intervention is the only curative treatment for RP. High recurrence and postoperative complication rates following RP repair are critical problems in various surgical treatments for RP. Therefore, we recommend choosing a surgical treatment for RP that has a high cure rate and low morbidity and mortality rates. Generally, it is accepted that trans-abdominal surgery has a lower recurrence rate and shows improved functional outcomes; therefore, it is preferred to trans-perineal surgery [1]; however, traditionally, surgeons choose the surgical procedure for RP based on their experience and preference. The risks of morbidity, mortality, and recurrence remain for elderly patients [11]. An ASA grade of 4 and the trans-abdominal approach were found to be risk factors for postoperative complications. In the report by Russell et al. [12], which evaluated morbidity and 30-day mortality in 1485 patients who underwent RP surgery in the American College of Surgeon’s National Surgical Quality Improvement Program between January 2005 and December 2008, the complication rates for the trans-abdominal and trans-perineal approaches were 12.9 and 7.6%, respectively. In addition, Lee et al. [13] reported that, in Asian patients, immediate major complications occurred more frequently using the trans-abdominal approach, including open rectopexy, than using the trans-perineal approach (25% vs. 15%), although a significant difference was not observed. On the contrary, Kaiwa et al. [14] reported that lap-rectopexy is as safe for patients aged > 70 years as it is specifically designed for younger patients. Recently, a systematic review by Emile et al. [15] reported that abdominal rectopexy for RP resulted in satisfactory improvements in symptoms and was acceptable in terms of the recurrence and morbidity rates; however, the trans-abdominal approach for RP remains controversial.

On the contrary, RP remains associated with a high recurrence following surgical repair. Ding et al. [16] reported that the recurrence rate following surgical repair for recurrent RP was significantly higher than that for primary RP. Especially for recurrent RP, an appropriate surgical procedure should be more carefully selected. Recently, some reports have suggested that a laparoscopic procedure could be an alternative to conventional abdominal procedures. Mustain et al. [17] reported no significant differences in the complication rates between the abdominal approach and perineal approach groups in a propensity-matched cohort. Additionally, de Bruijn et al. [7] evaluated the long-term outcomes of 80 patients with full-thickness RP, including 35 patients with recurrent RP, and stated that lap-rectopexy is a safe surgery for full-thickness RP. In this study of Japanese patients, the operative time was significantly longer in the lap-rectopexy group than in the perineal group; however, significant differences were not observed regarding blood loss or length of hospitalization between the two groups. Postoperative complications occurred more often in the lap-rectopexy group; however, no significant difference was observed between the two groups. In 34 patients (82.9%) in the lap-rectopexy group who had previously undergone trans-perineal repair, major complications did not occur. Most patients with recurrent RP could be treated safely with a more durable lap-rectopexy; however, cardiac and pulmonary postoperative complications were observed only in the lap-rectopexy group. The patient with a postoperative cardiac complication had a history of treatment for heart failure. Moreover, the patient with postoperative pulmonary complication had no history of treatment for pulmonary disease but was very old (aged 94 years). Both of these cases might be related to general anesthesia. Regarding recurrence, the overall recurrence rate following RP repair for patients with recurrent RP was 27.2% (21 patients), which was acceptable [8, 11]. In this study, 14 patients in the perineal group experienced recurrence. The recurrence rate of the perineal procedures was 28.6% in the Altemeier procedure, 50.0% in the Delorme procedure, and 21.4% in the Gant–Miwa procedure. Recurrence was mostly observed in the Delorme procedure in this study; however, the exact reason was not known. When the recurrence rates were compared between the two groups, the rate was significantly lower in the lap-rectopexy group, suggesting that lap-rectopexy is superior to the trans-perineal procedure for recurrent RP when patients can tolerate the surgery.

Additionally, we also evaluated the risk factors for recurrence following surgical repair for recurrence of RP in a multiple variable analysis. The known risk factors for RP include female sex, aging, chronic constipation and diarrhea, multiparity, and dementia, among others [18]; however, these potential risk factors, excluding female sex and aging, could not be evaluated because of small sample size in this study. Moreover, RP is a type of pelvic organ prolapse and is, thus, often accompanied by other pelvic organ prolapses. Catanzarite et al. [10] reported that pelvic organ prolapse significantly affected the recurrence rate following surgical repair for RP, although this was a retrospective study. Furthermore, de Bruijn et al. [7] noticed that a history of multiple previous prolapse repairs increased the risk of prolapse recurrence by 8.33-fold. However, in this study, these factors were not associated with recurrence following surgical repair for recurrent RP. Finally, lap-rectopexy was the only factor associated with low recurrence following surgical repair for recurrent RP.

We identified several limitations to this study. First, this was neither a randomized control study nor a propensity score matching study. Similarly, this study was not only retrospective in design but also had a small sample size. Second, in this series, the rectum was mobilized to the bottom of the pelvis laparoscopically, although the pelvic nerves were always preserved during surgery. Re-recurrence following rectopexy and symptoms, including constipation and fecal incontinence, following surgical repair, are reportedly associated with the level of dissection of the rectum during surgery [6, 7, 19]. We should have evaluated the functional outcomes, including long-term outcomes. Finally, there are various procedures—including mesh rectopexy, suture rectopexy, and ventral rectopexy—in lap-rectopexy. In this study, for all patients, laparoscopic suture rectopexy was performed without a mesh. Although some reports have suggested that laparoscopic suture rectopexy is a safe and feasible procedure compared to the laparoscopic mesh rectopexy [20, 21], we still need to further assess the difference in surgical outcomes and durability of the repair among various surgical procedures in prospective randomized multicenter trials.

## Conclusion

Our findings suggest that, in Japanese patients with recurrent RP, lap-rectopexy was an effective procedure with a lower recurrence rate than and a similar safety profile as a perineal procedure.
